# Die Technik der speziellen Therapie

**Published:** 1898-12

**Authors:** 


**Die Technik der speziellen Therapie.** Von Dr. F. GUMPRECHT. Pp. xi., 337. Jena: Gustav Fischer. 1898.

There is no English work on precisely the same lines as this. It contains descriptions of all operations which are practised in ordinary so-called medical—as distinguished from surgical—work; *e.g.*, excision of tonsils, intra-laryngeal operations, lavage of stomach, paracentesis of thorax and abdomen, lumbar puncture, bleeding, transfusion. It also has an account of catheterism and lavage of the bladder. The work is essentially a practical one; it describes in each case what is to be done and exactly how it is done, the difficulties and accidents which may be met with, and the results which may be expected to follow. The question of diagnosis is not entered upon. To each section is appended, in thorough German fashion, a good bibliography. The book is of great value and will be of considerable utility. It compares favourably with our "practical" books, which, for the most part, are written from an individualistic or simply English point of view, whereas this gives a *résumé* of the best from all sources.

